# Supplementary material for: Muscarinic modulation of M and h currents in gerbil spherical bushy cells
Source: PLoS One. 2020 Jan 15;15(1):e0226954. doi: 10.1371/journal.pone.0226954 (PMC6961914; doi:10.1371/journal.pone.0226954)
Supplement: Attachment — Submitted filename: Responses.docx [file pone.0226954.s001.docx]

**PD Dr. Thomas Künzel**

Principal Investigator

Worringer Weg 3

52074 Aachen

GERMANY

Building 5350

Floor 2, Raum Nr. 109

Phone:	+49 241 80-24854

Fax:	+49 241 80-22133

Mobile:	+49 151 18661207

kuenzel@
bio2.rwth-aachen.de

**14.10.2019**

**Revision of Research Article to PLOS One** **“Muscarinic modulation of M and h currents in gerbil spherical bushy cells” (PONE-S-19-21830)**

| **Bio2** \| Worringer Weg 3 \| 52074 Aachen \| GERMANY | 163310 |
| --- | --- |
| PLOS One  Editorial Board | |

Dear Editors,

herewith we submit the revised version of our manuscript “Muscarinic modulation of M and h currents in gerbil spherical bushy cells”. We addressed all editor and reviewer critique and have substantially revised the manuscript text, especially the statistical analysis and descriptions. We revised all figures and include now more new data (synaptic physiology and monitoring of physiological parameters in current clamp) than in the original submission. We thank the editor and the reviewers for the fair and constructive criticism and feel that the manuscript was considerably improved based on their input.

In the following we would like to give detailed response to the reviewers comments:

**Reviewer 1:**

1) The expression of M1/M2 receptors on SBCs has been investigated by immunohistochemistry and important conclusions are based on those data. However, the staining against M1 suggests a predominantly cytoplasmic localization, rather than membrane bound, which might be caused by unspecific binding of the antibody. Did the authors confirm the specificity of the antibody or performed the control experiment using an antibody that bounds another epitope (e.g. Alomone Labs Cat# AMR-001, RRID:AB_2039993 binds the third intracellular loop, rather than the C-terminus of M1 receptors)? Unfortunately, we did not try the M1 antibody from Alomone Labs Cat# AMR-001 or any other M1 antibody and we will not be able to perform this experiment at this time. However, we performed Western Blot with samples from gerbil hippocampus and auditory brainstem. A band was revealed around 75 kDa as expected and was weaker in the brainstem than in the hippocampus. Furthermore, controls were performed as in our previous paper: Gillet et al, J Comp Neurol, 2018.
2) The series resistance during ephys recordings was not compensated, but the authors do not report Rs values during the recordings or if there were any differences between groups or changes over time. While the influence on the slow and rather small IM currents might be negligible, a high Rs will likely affect the fast, larger IInst currents which in turn could affect the analysis of IM. Likewise, the amplitude and kinetics of AP-evoked EPSCs (Figure 7) will critically depend on the recording’s Rs due to the non-linearity of Rs compensation. Unless the uncompensated Rs values during recordings were either small (<5 MOhm) or very similar between groups this could introduce considerable errors when estimating amplitude and kinetics. The authors should therefore also report the Rs values for the individual recordings or declare if recordings with high Rs (e.g >>10 MOhm) were discarded. For each experiments, we indicate the mean Rs and stated the Rs criterion we used in the methods to determine if we keep or discard the recording from a given cell.
3) The authors recorded from P14-P30 gerbils of either sex. In their previous study (Gillet, 2018,J. Comp. Neurol.), the authors reported an increase in cholinergic innervation between P15 and P28 and another recent study (Mueller et al, 2019, Front. Cell. Neurosci., DOI: 10.3389/fncel.2019.00119) suggest that some properties of mouse CN neurons develop after hearing onset. It is not clear why the age range was chosen so close to the animal’s hearing onset and no information is provided on the age distribution for the different experiments (except for immunohistochemistry at P30). Considering the contrasting effects of oxoM and 4DAMP in some of the recordings (Figure 9, some cells show increase in RMP, other decrease), did the authors split the data set by age or sex to determine if those factors might account for the observed variability, e.g. activation of mACh might be depolarizing in young, but hyperpolarizing in older animals or vice versa. Since the authors have those data readily acquired, it might be worth adding a statement, if any difference in sex/age was observed. If a certain age range was used for some of the recordings for experimental reasons (e.g. recordings more stable), this should be mentioned. We added the range of age of the animals we used for each experiments. It is true that P14 is close to hearing onset (P12), however it is difficult to get good recordings at ages older than P25 that is why we used gerbils aged from P14 to P25 for electrophysiological recordings, stated now in the revised manuscript. As mentioned in Gillet et al (2018), the cholinergic innervation is already present in every part of the AVCN at P15, it is therefore safe to assume that muscarinic receptors are already expressed and functional as this age. Moreover, no correlation was seen between the response type and the age. A paragraph mentioning age as a factor for variability was added to the discussion section.
4) Based on fiber stimulation and mEPSC recordings with application of carbachol, the authors conclude that cholinergic transmission does not affect synaptic transmission at SBCs. However, the authors also show that application of 4DAMP (an M3 antagonist) hyperpolarizes SBCs, suggesting a tonic activation of M3 receptors in the slice. If ACh receptors were tonically activated at the endbulb of Held, the effect on synaptic transmission would likely be negligible when using ACh agonists but would require the use of ACh antagonists. This possibility should be tested or at least discussed. Thank you for pointing this out. We mentioned these considerations in the revised discussion.
5) The authors performed fiber stimulation at 100 Hz and analyzed the EPSC kinetics for the first EPSC. Did the authors also test higher (e.g. 300 Hz) stimulation rates, which would more closely mimic in vivo activity? A potential ACh modulation of synaptic vesicle release might be more pronounced during ongoing activity. Similarly, the authors analyzed EPSC kinetics for the first EPSC only. Since the first EPSC after a period of quiescence is somewhat artificial (considering the high spontaneous rates of most auditory brainstem neurons), it might be worthwhile to also analyze the EPSCs later in the train (if the signal-to-noise ratio permits such analysis).

We also recorded (but did not analyze for the first version of the paper) 200Hz and 50Hz stimulation rates. These result we now also present in the revised version of the manuscript.

Concerning the question about the rise and decay kinetics later in the train: this is an interesting aspect we do not usually analyze, mostly due to signal-to-noise concerns. We tried this in our present data and reanalyzed kinetics for evoked EPSC recordings for EPSC_2_ up to EPSC_15_. No statistically significant differences were seen either.

This finding and further new synaptic analysis (asynchronous release ratio & release probability; no significant differences) are now shown in the revised Figure 7. Appropriate methods descriptions and results were added to the manuscript body.

6) Application of the mACh receptor agonist oxoM hyperpolarized the SBC and the authors argue that an indirect block of Kv7 channels causes this effect. While this argument is conceivable and has been shown in other systems, the authors should be aware that oxoM can directly block Kv7 channels without the activation of mACh receptors (see Zwart et al., 2016, Eur. J. Pharm. DOI: 10.1016/j.ejphar.2016.08.037). Did the authors perform control experiments with oxoM + atropine to block mACh receptors or used a more specific mACh agonist (e.g. oxotremorine or xanomeline) which has been shown to not interact with KCNQ channels? If not, the authors should at least discuss this possibility and how it might affect their conclusions. We did not perform control experiments with oxoM+atropine or used a more specific agonist. This possibility is now discussed in the revised paper.
7) The model in Figure 10 proposes two opposing effects of endogenous ACh, one leading to depolarization by Kv7 closure, the other to hyperpolarization by HCN closure. The authors speculate that brief ACh application will predominantly bind to M1/M3 receptors (highly expressed), thereby depolarizing the cell, while during prolonged application the hyperpolarization by M2 (weakly expressed and located at the neuropil) activation dominates. While this explanation is reasonable in the current experimental setting, it might not apply in vivo (since the origin and activation dynamics of those inputs are barely known). The authors should discuss if either of those conditions might be favorable during varying acoustic stimulation (e.g. hearing in noise, natural sounds). We try to address this point now in the revised discussion section.
8) The proposed model suggests the involvement of secondary messengers (cAMP, PLC etc) to act on Kv7 and HCN channels. While this is reasonable and has been shown in other systems, I wonder if some of the reagents have been washed out/diluted over time in the whole-cell experiments, thus underestimating the observed effect. This could be tested by e.g. gramicidin-perforated patch-clamp experiments. Do the authors have any data on this? If not, new experiments are not required, but the authors should acknowledge and discuss this possibility. No, we unfortunately did not perform any perforated-patch recordings. We mention these considerations to the reader in the discussion section.
9) Line 502: “oxoM induces closure of M channels […] and depolarizes the resting membrane potential”. This conclusion is not immediately evident from the data, since application of oxoM (Figure 9G) resulted in an, on average, small hyperpolarization. The authors might argue that block of M3 channels hyperpolarized the cell, but unless M1/M3 channels were specifically activated and result in depolarization, this statement is too strong given the underlying data.

We removed this statement.

10) Previous studies by the corresponding author and others (e.g. PMIDs: 21411667, 25164657, 28945194, 27855778, 23345233) point to an important role of inhibition in shaping SBC responses, particularly during complex or naturalistic acoustic stimulation. Did the authors find any evidence for ACh receptors on inhibitory inputs contacting the SBCs?

Most recordings were performed in the presence of blockers of inhibitory transmission in this case so no physiological data are available. We also did not perform any co-staining experiments to address this. The reviewer however raises an interesting point that would warrant a dedicated study. We mention this briefly in the discussion section now.

11) Slices were incubated at room temperature and all recordings were performed at room temperature and at supra-physiological calcium (2 mM vs. 1.2-1.5 mM). Since the kinetics of G-protein mediated signal transduction pathways are temperature and calcium dependent, the observed effect in the experiment might be different to in vivo conditions, especially considering the proposed “balance” between M1/M3 (depolarizing) and M2 (hyperpolarizing) mediated pathways. While I acknowledge that the authors performed similar experiments at 37C in a previous publication (Figure 4 in Goyer et al, 2016, eNeuro), it might be worth discussing the effect of temperature and calcium concentration in the light of the two proposed pathways.

We mention this to the reader as a caveat in the discussion section now.

12) The manuscript would benefit from a statistics section within the Methods. Although the statistics for this data set is mostly straight forward, the authors should describe how sample size and statistical tests were selected (e.g. testing for normal distribution, etc.).

Statistics sections inserted, also in the light of the new statistical analysis we now performed for the revised paper.

13) Line 98: Please state how the junction potential was estimated, by calculation or experimentally. Was the junction potential also corrected for the voltages shown in Figure 9B? The junction potential was calculated, which is now indicated in the revised manuscript. As written in the methods, the junction potential was only corrected for I_M_ and I_h_ measurements thereby not corrected for any results shown in Fig 9, 9B included.
14) Line 156: Please state the concentration of Triton X-100 in PBS. Was it 0.1 % as in the subsequent steps?

Added the concentration (0.1%)

15) Line 79: Please indicate which temperature was considered room temperature (e.g. 25C). Added
16) For antibodies and software used, please include RRIDs where available (e.g. line 192 “rabbit anti-M1 muscarinic receptor (Alomone Labs Cat# AMR-010, RRID:AB_2340994)”). Added
17) In the Methods, the authors describe that cells were filled with biocytin during recordings, but its purpose appears unclear, since the immunohistochemistry was performed on animals not used during slice recordings. If the biocytin staining was used to morphologically verify the cell type after the end of recordings (e.g. to identify SBC vs. stellate cells), it should be stated in the Methods and Results section. Added
18) Line 143: Please indicate how many orders were used for the polynomial fit and if any constraints were used. This information was added to the method section.
19) The manuscript frequently reports p-values as p=1. While I understand that p-values may be >1 after applying the Bonferroni correction on the actual p-values rather than the alpha value of the test, it is better to report such p-values as p>0.9999, since p=1 would only be possible if the means of two groups are identical. Corrected
20) Figure 1: In the bottom part of Panel A, the response to multiple depolarization steps is shown, but the indication of IM, ISS etc. is for one of the traces only, which might be confusing. Consider indicating for which trace the labels apply, e.g. using different colors or line styles. We made the changes of the figure 1. Now the step used to identify the M current and its legend are in black while the rest is grey.
21) Figure 1: Consider replacing the title of Panel C “IM at -50mV” with “Determination/Identification of IM at -50mV” or alike, since the panel shows all currents, not only IM. Corrected
22) Figure 2: The panel organization is hard to follow. Consider moving the current-voltage graphs next to the corresponding traces. Also, it appears that the same data is labeled “XE991” in panel D and “ctrl” in panel G. While I understand the rationale of doing so, it might be clearer to combine those two panels into a single graph (labeled as ctr, XE991, XE991+oxoM which is then consistent with the raw traces). It might also be easier to follow, if the y-labels of panels D/F/G were labeled “Inward relaxation current IM”, as to link it to IM in panel C. We revised Fig 2 as suggested (as well as Fig 4 to have the same organization).
23) Figure 2: The y-labels on panels D/F/G is not centered on the y-axis. We corrected it.
24) Figures 3-5: Please be consistent regarding the nomenclature of the currents. For instance, in Figure 3A-C the current is labeled “inward relaxation current”, but labeled “IM” in panel D. In my understanding those are the same currents but using different names is potentially confusing. Similar in Figures 4/5 with most panels labeled “inward current”, but “inhibition of Ih” in Fig. 5D. In my understanding, the term “inward current” is incorrect in this context, since it only refers to Ih, not the total inward current (as indicated in Figure 4C). We corrected the labels to be consistent in all figures.
25) Figure 3 & 5: The currents at -50 mV (Figure 3) and -110 mV (Figure 5) were analyzed using paired t-tests. Since multiple measures have been taken from the same cells (ctr vs. drug and at different voltages) I feel that a two-way repeated measures ANOVA would be more appropriate and informative. In this case, the data should be analyzed using the within-subject factors treatment (ctrl vs. drug) and voltage.

We performed the analysis as suggested by the reviewer. We state the approach in the method section and report the statistics and results in the result section at the appropriate positions. The description of the panel in the figure caption were adjusted as well when necessary.

26) Figure 6: The DAPI labeling looks unusual and different from previous papers from the same authors (e.g. Gillet et al, 2018, J. Comp. Neurol.), i.e. nuclei are barely labeled (see the “holes” in the cells in panel D, which should contain the cell nuclei) and most of the labeling is constrained to the auditory nerve root. Since the DAPI labeling is not critical for the conclusion (and not referred to in the manuscript), consider removing this channel from the images. We removed the DAPI staining in Fig 6.
27) Figure 6: Please include which magnification (e.g. 60x) was used for imaging. We indicated the magnification in the figure caption.
28) Figure 8: Since the detection of mEPSCs is highly variable and critically dependent on threshold selection, it would be helpful to indicate detected mEPSCs in the representative traces shown in panel A, so as the reader has a better understanding of which events were considered mEPSCs. Red dots were added to the trace to indicate the detected mEPSCs.
29) Figure 8 & lines 395-404: The data are analyzed using ANOVA, but not details are provided as to which type of ANOVA was used. Given the experimental design, it seems a one-way repeated measures ANOVA would be appropriate. Please indicate this in the text.

We now include more information about the statistics and the result of the used tests (one-way repeated measures ANOVA) in the text at the appropriate locations.

30) Figure 9: Please rescale the y-axis in panel H bottom to avoid cutting off data points. Corrected
31) Figure 10: Please label the small red circles as “Acetylcholine”. Added
32) Line 223: “Array of blockers” awkward wording, perhaps replace with “combination/cocktail of blockers”. Corrected
33) Line 360: consider replacing “bigger M2 signals” with “intense M2 signals”. Corrected
34) The authors should consider replacing the bar charts in Fig. 7/8 with box plots but keeping the individual data points to better display the data distribution. We agree with the reviewer that there should be more emphasis on the individual data points and thus we replaced the bars in the chart by a mean +/- std marker.
35) Lines 465-475: The authors explained that when excluding parts of the data set (cells with no or depolarizing effect) the statistical tests became significant. While this is not surprising (even a normal distribution with enough N will show “statistical significance” when limiting the data set to only positive or negative values), it implies that those excluded cells “prevented” statistical significance. Unless those cells were excluded based on criteria (e.g. age, sex, recording quality etc.) other than their response to drug application, this exclusion seems unjustified and potentially misleading. The authors did a good job in pointing out the differences in responses but should probably remove the statistics of the partial data set (after exclusion of those cells). We removed the statistics made after grouping of cells.
36) Line 498: “For the first time […] IM was shown in SBCs”. This statement is too strong considering that Marx & Manis, 1991 (J Neurosci, DOI: 10.1523/jneurosci.11-09-02865.1991) reported a very similar low-threshold conductance and originally discussed the presence of an M current in bushy cells. We reformulated this sentence accordingly.
37) Line 515-416: unclear what is meant by “other synaptic functions”? The authors did not test the effect of cholinergic transmission on e.g. LTP or homeostatic plasticity, so this statement is vague and should be rephrased. We replaced “other synaptic functions” by “short term plasticity”.
38) Lines 26 & 564: Consider removing/replacing the subjective term “interestingly”. Removed
39) Line 143: typo in “substracting” (remove second “s”). Corrected
40) Line 149: replace “glycinergic and GABAergic blockers” with “glycine and GABA receptor blockers”. Corrected
41) Line 374: replace “immunohistocheminal” with “immunohistochemical”. Corrected
42) Line 424: “cell conductance” should probably read “cell capacitance”. Yes indeed, change made.
43) Line 430: replace “showed in (A)” with “shown in (A)”. Corrected
44) Line 569: missing “t” in “spontaneous”. Corrected
45) Line 539: please explain what “nACSF” stands for, if the additional “n” is not a typo. Corrected
46) Line 540: replace “a HCN blocker” with “an HCN blocker”. Corrected

**Reviewer 2:**

1) The manuscript needs some grammar and language polishing. Some sentences seem incomplete or awkward. For instance: pg 12, ln 273 “in control conditions of by the …”.

We revised the language throughout the manuscript.

2) In some cases (Fig. 2 legend), the description of the data is too succinct. We tried to improve the data description in several instances (also Fig 2 caption).
3) Although the authors do explain that they use whole cell recordings to identify the muscarinic receptor subtypes and the currents involved in spherical bushy cells, and this is indeed very interesting an sound, the manuscript would benefit from a clearly stated hypothesis. We added a sentence to the introductory section (l.46)
4) The age range of the animals is 14-30 days. While 14 days is juvenile, 30 days is considered as young adult, which may lead to increased variability of the results. Also, the expression levels of KCNQ and HCN channels is age dependent (see (Buskila et al., 2019) and should be discussed. A paragraph about this topic was added in the discussion.
5) It is unclear which protocol was used to measure Ih? How can it be the same as for Im (P6)? Please explain. The M current is an outward current but can be measured by the slow inward relaxation corresponding to the closure of M channels while I_h_ is an inward current corresponding to the opening of HCN channels. What we meant is that both currents can be measured by applying hyperpolarization steps but at different range of voltages. We have clarified this in the method by describing the protocol to measure I_h_ independently of the I_M_ protocol.
6) Please explain why you chose the concentration of Carbachol, as 500uM seems a bit high. In our previous paper (Goyer et al, 2016) we already used a concentration of 500uM (puff application). Other studies (see for example Fujino and Oertel, 2001) used similar concentrations (both 100 and 500uM) to show cholinergic effect on AVCN stellate cells.
7) Fig 1. I’m not aware about all the plethora of K+ channels expressed in BSC, but the fact that one can see an outward current not necessarily imply that this current is Im. The authors should add a selective blocker to confirm the identity of this current, as done in fig 2.Also, I’m not sure why fig. 1 and fig. 2 are not joined. I don’t see any added value for both figures.

We would like to keep figure 1 as it in our opinion greatly helps to clarify the experimental approach. Apart from that, we do not really know how to comment further on this. The current we measure (as shown in Fig 1) is pharmacologically confirmed to be M current. The reviewer is correct that the simple existence of an outward current does not indicate M current yet, since high- and low-threshold potassium currents are also present in these neurons. However, those prominent potassium currents have much more rapid kinetics (Kv1 and Kv3 type channels) or significantly inactivate over time (A type currents). The stimulus protocol (which is explained in Figure 1!) is designed to distinguish currents based on these characteristics. Furthermore we’d like to add that the M current was named, because it is a voltage-activated current that has the speciality of being modulated by muscarinic activation. The fact that both XE991 (a specific blocker) and oxotremorine (the modulator) affect the current makes us reasonably confident that this current is an M current. We explain this reasoning to the reader in more detail now in the result section (l.236ff)

8) Fig. 3d – is not clear. Please re-label the graph to clearly identify the difference between XE991ctrl and XE991, OxoM-xe991 and OxoM ctrl. We changed the labels in the figure and the text.
9) Fig. 9E legend– perfusion of what? nACSF? What is it, not explained in the text. Replaced by ACSF.
10) Fig. 9G – the current data show no significant difference if all data is included and becoming significant if some data is excluded. If there is a good reason for excluding data, please do so. If the authors convinced that there is a trend, they should increase the n-number. We cannot increase the N number since more experiments cannot be performed at this moment, but we decided to remove the statistics once some cells were excluded and only mentioned that some cells acted at the opposite of the average effect. See response to comment 35 of reviewer 1.
11) Statistical tests – It is not clear which ANOVA was used? One way, two way? What was the significance level and df? These should be clearly stated. The significance level is now stated in the statistics part in methods. The exact statistical test and df are now specified. See also comments by reviewer 1.
12) Oxotremorine-M potentiate NMDA currents; however, the authors seems to neglect this. How can it affect the results? Ca2+ and Na+ entry? The glutamatergic currents at the endbulb of Held after hearing-onset are predominantly mediated by AMPA receptors (Walmsey, 1998). Application of only CNQX/DNQX was sufficient to fully suppress e/mEPSC in our experiments. We did not think that this specific side-effect of OxoM played a significant role in this study. We nevertheless mention these considerations to the reader in the revised discussion section.
13) Immunohistochemistry data– how many slices were examined? No stat is reported. The total number of animals used for immunohistochemistry and the number of slices used for each antibody is now stated in the methods.
14) Although the authors looked into the impact of Im and Ih on the subthreshold activity in BSC (RMP, IR), the suprathreshold physiological activity (firing rate, resonance, SFC’s) was not tested. If the authors have the data, they should present it as it would strengthen this study findings. We tested the effect of cholinergic modulation on spike probability before (Goyer et al., 2016). Since M-currents are sometimes implicated in adjusting the AP threshold we analyzed this metric here as well now (appropriate text in the method and result section was added). We also added a quantification of the “voltage sag” (i.e. the effect of the hyperpolarization activated channels) and show that this is reduced by the muscarinic activation (as expected). Both new analyses are presented in the new figure 10 (+caption). The old figure 10 (the summary scheme) is now figure 11.
15) Minor issues Pg 3, Ln 39 – please identify VCN as the ventral cochlear nucleus. Done
16) Pg 3, Ln 40 – please identify SBC. Done
17) Pg 6, Ln 115 – a 10 current steps protocol should state the range of injected currents. We indicated the range of injected currents.

Kind regards,

Dr. Thomas Kuenzel
