# Supplementary material for: Muscarinic modulation of M and h currents in gerbil spherical bushy cells
Source: PLoS One. 2020 Jan 15;15(1):e0226954. doi: 10.1371/journal.pone.0226954 (PMC6961914; doi:10.1371/journal.pone.0226954)
Supplement: Attachment — Submitted filename: Response_to_Reviewers.docx [file pone.0226954.s002.docx]

**PD Dr. Thomas Künzel**

Principal Investigator

Worringer Weg 3

52074 Aachen

GERMANY

Building 5350

Floor 2, Raum Nr. 109

Phone:	+49 241 80-24854

Fax:	+49 241 80-22133

Mobile:	+49 151 18661207

kuenzel@
bio2.rwth-aachen.de

**05.12.2019**

**Revision of Research Article to PLOS One** **“Muscarinic modulation of M and h currents in gerbil spherical bushy cells” (PONE-D-19-18119R1)**

| **Bio2** \| Worringer Weg 3 \| 52074 Aachen \| GERMANY | 163310 |
| --- | --- |
| Andrea Barbuti, PhD  Academic Editor  PLOS ONE | |

Dear Editors,

herewith we submit the additional revisions of our revised manuscript “Muscarinic modulation of M and h currents in gerbil spherical bushy cells”.

We again thank the editor and the reviewers for the fair and constructive criticism.

Kind regards,

Dr. Thomas Kuenzel

In the following we provide a point-by-point list of reviewer comments and our responses:

**Reviewer 1:**

1. Line 334: “OxoM” should probably be lowercase. Corrected.
2. Line 364: “washing” should perhaps read “wash-in”. It should. Corrected here and in a few other instances.
3. Figure 7C: The 200Hz condition seems to show the mean values only with individual cells missing. In fact the individual cells all show asynchronous release values of, or very close to, 0.5, so they are all hidden by the solid marker. We point this out now in the caption.
4. Figure 10A: The Figure legend (Line 600f) describes the right panel as “after 10 min of oxoM perfusion” but the figure panel is labeled “recovery” (i.e. some time after drug application has been stopped). It should read “10min after”. Changed the wording accordingly.
5. Figure 10A: Please label the middle panel “oxoM wash-in”, since multiple drugs (carbachol, oxoM, 4DAMP) have been used in the figure. Changed.

**Reviewer 2:**

1. Although the authors corrected many grammatical errors, I suggest a further editing and proof reading to aid clarity and make the text more readable. We tried to further improve the grammar and the readability of the manuscript.
2. Moreover the affiliation of the manuscript was a bit awkward as the figure legends were mixed into the text, without the figures, which to my opinion just adds to the confusion. I fully agree. This is however formatted as suggested under “Manuscript organization” in the submission guidelines. It says: “Figure captions are inserted immediately after the first paragraph in which the figure is cited. Figure files are uploaded separately.”
3. In the methods section, the authors state that they chose to include cells with quite high Rs (up to 35 MOhm) and allowed fluctuations of 30%. All these parameters can significantly affect their recordings and therefore conclusions. As the authors state that no more experiments can be made, I suggest commenting on this in the discussion. We mention this fact in the discussion now.
4. Ln 138. Please rephrase to “One way ANOVA with repeated measures”, as well as other places in the text. We were under (wrong?) the impression that “one-way repeated measures ANOVA” was the standard term. Changed all instances nevertheless.
5. Results section, ln 286 onwards: this section is written like a figure legend, which is quite repetitive, why? We removed the two “figure legend”-like sentences.
6. Results in Fig 3A are a bit odd. The plot shows that there is almost no difference between the data points at -70mV, yet its deemed significant as there is a small Im current at this voltage? That is indeed what it is going on here. The difference is 9,1 pA with a standard error of ±2,8pA, which results in p=0.027 [the confidence intervals 1,69-16,58 do not include 0, thus the difference is statistically significant]. The p value of 0.027 is below our “one-star” criterion of p<0.05). Our interpretation is, that there is a small Im current already activated at -70mV that gets blocked by the XE991 compound, yes.
7. Fig. 3D – maybe I’m missing something, but in the legend the authors state that the comparison was between the treatment to control conditions (no treatment), yet in the plot it seems like the authors compared between the different treatments. Please clarify. Also, does the results with OXOM+XE991 means that there was no inhibition of Im, or that compared to XE991 alone, there was no additional inhibition? This needs clarification! We improved the clarity of these statements in the caption and the results paragraph.
8. The description of the measured currents (Im, Ih) repeat itself too many times ( methods, results and legend). Removed repetitive descriptions.
9. Ln. 385 – the conclusion that “ The presence of ZD7288 before oxoM application prevented large effects of oxoM on Ih” does not make any sense, as in the presence of ZD7288, there is no Ih, so where this expectation came from… Reworded.
10. Ln 409: the overall conclusion is wrong. Essentially, all you show is that cholinergic agonist decrease the Ih, there is no evidence to support the conclusion that Ih play a role in the cholinergic modulation of SBC’s. We reworded the conclusion to say: “**This suggests** that both IM and Ih **could potentially** play a role as effectors in the cholinergic modulation of spherical bushy cell”, which is hopefully more conservative.
11. Fig 10 legend: the plots in A are not 10 current steps (as only two traces are shown). Moreover the labels in the plot suggest “wash-in” and “recovery”, while the legend suggest it is 3 min and 10 min post carbachol application. I think there is a mix-up with fig 9A, Please clarify. Label and legend description was corrected (also see comment 4 by reviewer 1).
12. I found result 10J a bit puzzling, and essentially contradict Fig 4f. So did OXOM decrease Ih or not? The net effect on Ih averaged over all cells is indeed small, which we cannot fully explain given the robust effect shown in Fig 4. However please note: the results from the current clamp experiments shown in 10J do not contradict the results in Figure 4f! Figure 10J shows a reduction of the voltage sag amplitude, as in: the difference in mV between hyperpolarization at the beginning of the current injection and the steady state hyperpolarization is smaller under oxoM – this is well in line with the reduction of Ih current. We try to make this reasoning more clear in the result section for the reader.
